# Nosocomial Outbreak of Drug-Resistant Streptococcus pneumoniae Serotype 9V in an Adult Respiratory Medicine Ward

**DOI:** 10.1128/JCM.02405-16

**Published:** 2017-02-22

**Authors:** Elita Jauneikaite, Zareena Khan-Orakzai, Georgia Kapatai, Susannah Bloch, Julie Singleton, Sara Atkin, Victoria Shah, James Hatcher, Dunisha Samarasinghe, Carmen Sheppard, Norman K. Fry, Giovanni Satta, Shiranee Sriskandan

**Affiliations:** a Health Protection Research Unit in Healthcare Associated Infections and Antimicrobial Resistance, Imperial College London, London, United Kingdom; b Department of Medicine, Imperial College London, London, United Kingdom; c Imperial College Healthcare NHS Trust, London, United Kingdom; d Respiratory and Vaccine Preventable Bacteria Reference Unit, Public Health England, National Infection Service, Colindale, London, United Kingdom; e North West London Health Protection Team, Public Health England, London, United Kingdom; f Centre for Clinical Microbiology, University College London, London, United Kingdom; University of Iowa College of Medicine

**Keywords:** Streptococcus pneumoniae, nosocomial infections, bacteremia, pneumonia, serotype 9V

## Abstract

Streptococcus pneumoniae infections arising in hospitalized patients are often assumed to be sporadic and linked to community acquisition. Here, whole-genome sequencing was used to demonstrate nosocomial acquisition of antimicrobial-resistant sequence type 156 (ST156) serotype 9V S. pneumoniae in 3 respiratory patients that resulted in two bacteremias and one lower respiratory tract infection. Two of the cases arose in patients who had recently been discharged from the hospital and were readmitted from the community. Nosocomial spread was suspected solely because of the highly unusual resistance pattern and case presentations within 24 h of one another. The outbreak highlights the potential for rapid transmission and the short incubation period in the respiratory ward setting.

## INTRODUCTION

Streptococcus pneumoniae (or pneumococcus) is recognized as the main bacterial cause of community-acquired pneumonia presenting to hospitals (1). Pneumococcal carriage is considered to be a predisposing factor for pneumococcal infection; it takes from a few days to a month after acquisition of the pneumococcal strain to develop invasive pneumococcal disease (2). As the pneumococcus is asymptomatically carried by 10% to 30% of adults (3, 4) and can be carried by 30% to 86% of children (4, 5), cases arising in patients who are already hospitalized are often assumed to represent sporadic infection stemming from earlier acquisition in the community. Nonetheless, sporadic clusters and outbreaks have been reported where vulnerable individuals are in close proximity or contact with other people, such as specialized hospital wards (6, 7), elderly care centers (8, 9), nurseries (10, 11), and military camps (12).

Here, we describe a cluster of three cases of pneumococcal infection that arose within a period of 6 days in an acute care setting. Nosocomial transmission was suspected solely because of very recent hospitalization and identical but unusual antimicrobial resistance patterns and was confirmed by whole-genome sequencing (WGS).

### Outbreak cases.

**FIG 1:**
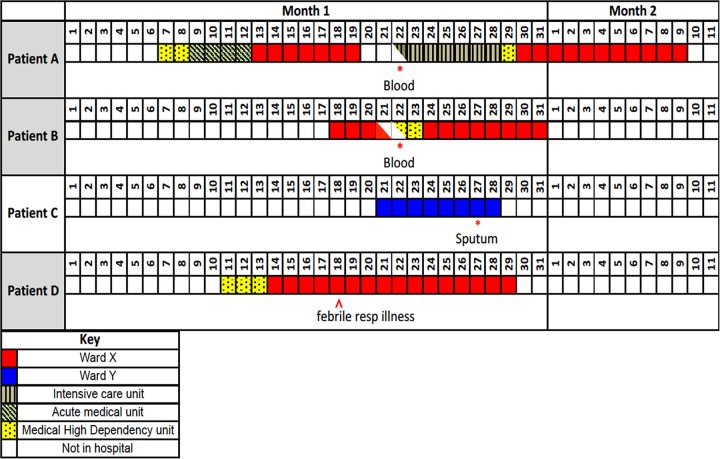
Timeline of confirmed cases of invasive S. pneumoniae. Timeline of patients A, B, C, and D in relation to their admissions, discharges, and onset of confirmed pneumococcal infection. Each small box indicates a single day. Numbers above boxes indicate consecutive days in calendar months; * indicates positive S. pneumoniae culture from a clinical sample.

Patient A, a 60-year-old female, was admitted severely unwell with sepsis and pneumonia. Blood cultures yielded S. pneumoniae, with intermediate susceptibility to penicillin but resistance to macrolides and tetracycline. The patient required intensive care unit (ICU) support. Three days prior to this presentation with pneumococcal bacteremia, she had been discharged from the same hospital following a severe noninfective exacerbation of asthma (Table 1), where she was managed initially on the medical high dependency unit (HDU) and then on ward X. Her first admission lasted a total of 13 days, 7 of which were spent on ward X (Fig. 1).

**TABLE 1:** Clinical details of patients affected by the nosocomial outbreak of S. pneumoniae

Patient	Days post 1st pneumococcal-positive case	Age (yrs)	Gender^*a*^	Source of pneumococcal isolate	Associated pneumococcal disease^*b*^	Year of pneumococcal vaccination	Underlying condition	FEV1 (%)^*c*^	FVC (%)^*d*^	Antibiotic (prophylaxis)	Previous antibiotic	Steroids at admission (dose)
A	0	60	F	Blood	Pneumonia, sepsis	2000	Asthma	NA^*e*^	NA	Ceftriaxone	None	Budesonide inhaled, prednisolone (20 mg/day)
B	0	76	F	Blood	Pneumonia, sepsis	1998	Asthma, diabetes	0.68 (63)	0.73 (53)	Ceftriaxone	Amoxicillin	Beclomethasone inhaled, prednisolone (4 mg/day)
C	6	73	M	Sputum	Pneumonia	2008	COPD	0.79 (29)	2.07 (58)	(Levofloxacin)	Doxycycline	None
D^*f*^		59	F	NA	None	Unknown	COPD, home NIV^*g*^	0.66 (27)	NA	(Levofloxacin)	Meropenem	Prednisolone (25 mg/day)

a F, female; M, male.

b Pneumonia was confirmed with chest X-ray for patients A and B and CT scan for patient C.

c FEV1, forced expiratory volume in 1 s (% predicted).

d FVC, forced vital capacity (% predicted).

e NA, not available.

f Patient D (close contact of patient C and on the same ward as patients A and B) did not have pneumococcal disease identified but had an acute febrile illness prior to pneumococcal isolation from patients A, B, and C.

g NIV, noninvasive ventilation.

Patient B, a 76-year-old female, presented with fever and breathlessness to the emergency department within 24 h of a recent discharge from the same hospital. Her presentation was also within 24 h of patient A's presentation to the same emergency department with pneumococcal bacteremia. Patient B's blood cultures were also positive for S. pneumoniae, with intermediate susceptibility to penicillin but resistance to macrolides and tetracycline. Patient B initially required high dependency (level 2) care. Prior to her admission with bacteremia, patient B had also been a patient on ward X with an infective exacerbation of asthma complicated by congestive cardiac failure and was treated with oral amoxicillin (Table 1). Patient B's stay on ward X, however, overlapped with that of patient A by less than 48 h (Fig. 1).

Patient C, a 73-year-old male, was admitted to a different ward (ward Y) with a collapse and infective exacerbation of chronic obstructive pulmonary disease (COPD); thus, he received empirical treatment with doxycycline (Fig. 1 and Table 1). Although a chest X-ray did not reveal overt consolidation, a computed tomography (CT) scan of the thorax demonstrated bibasal consolidation and a spiculated lesion in the left lung base. A sputum sample that was collected 6 days after admission yielded S. pneumoniae that was intermediate to penicillin and resistant to macrolides and tetracycline. Notwithstanding this result, the patient improved with supportive care.

Prior to his admission, patient C had been regularly visiting his wife, patient D, who was an inpatient on ward X with end-stage COPD. Patient D's admission overlapped with the first admissions of both patient A and patient B (Fig. 1). Patient D had negative sputum culture, and inflammatory markers were not raised when first admitted. However, 1 week after admission, she developed an acute febrile illness with cough and worsening shortness of breath and an acute rise in C-reactive protein to over 300 mg/liter. Microbiological samples were not sent, although her inflammatory illness responded rapidly to an empirical 5-day course of meropenem.

Patient D's febrile illness occurred 3 days prior to the admission of her husband (patient C) and 4 days prior to the readmissions of patients A and B with pneumococcal bacteremia (Fig. 1). Both patients A and B were on the same ward as patient D during patient D's febrile illness; patient B and patient D were in adjacent beds. Patient D made a sufficient recovery for eventual transfer to a hospice for ongoing palliative care. Patients A, B, and C made full recoveries from their acute illnesses and were discharged home.

## RESULTS AND DISCUSSION

All three pneumococcal isolates (PHESPV1509, PHESPV1510, and PHESPV1524) from the cluster were serotype 9V and had indistinguishable antibiotic susceptibility patterns; they were intermediate to penicillin (MIC, >0.5) and resistant to macrolides (erythromycin MIC, >16) and tetracycline (MIC, >8) (see Table S1 in the supplemental material), highlighting a potential outbreak. We considered whether laboratory contamination may have explained the findings; however, the individual blood cultures were processed entirely separately in a centralized laboratory using the Bactec system for blood cultures, and sputum cultures were processed in a designated containment level 3 (CL3) area making cross-contamination between any of the blood or sputum isolates highly unlikely.

An outbreak investigation identified a total of 12 close contacts of the affected patients from ward X. Chemoprophylaxis with 3 days of levofloxacin and pneumococcal vaccination with 13-valent pneumococcal conjugate vaccine (PCV13) or 23-valent pneumococcal polysaccharide vaccine (PPV23) were offered to any contact of patients who could be traced within 14 days of exposure, whether in the hospital or in the community; this included patients C and D. For practical reasons, contacts and health care workers (HCW) were not screened for carriage of S. pneumoniae. HCW were offered advice about invasive pneumococcal disease but were not given prophylaxis. No further cases of serotype 9V arose. It was concluded that the source of infection was likely to have been either patient C or patient D (who may have been further linked by the possible sharing of oxygen and nebulizer equipment at home), as both were present on ward X, either as inpatient or visitor, during the admissions of patients A and B; however, an alternative common source in ward X could not be ruled out.

**FIG 2:**
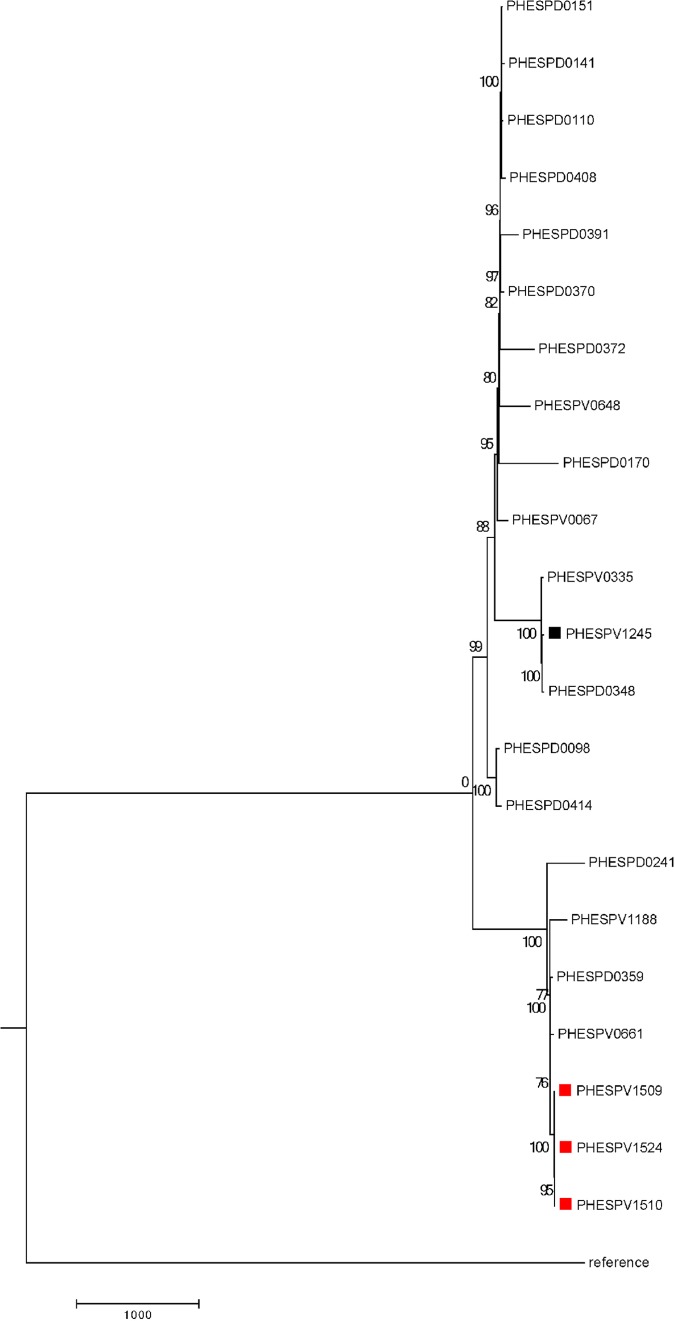
Neighbor-joining phylogeny tree of UK ST156 serotype 9V strains showing outbreak strains. SNP-based phylogeny tree of ST156 serotype 9V strains showing three outbreak strains and 19 contemporary strains (see Table S1 in the supplemental material). The branch numbers show bootstrapping, whereas the branch length is indicative of the SNP distance. Outbreak isolates are indicated with red squares. A black square indicates an isolate received earlier in the same year from the same region as the outbreak. The reference used was Streptococcus pneumoniae R6 (NC_003098). The average SNP distance between the outbreak strains and contemporary strains was 703 SNPs, with an average of 8,620 SNPs between the outbreak strains and the reference strain.

The initial examination of WGS indicated that each of the outbreak isolates was sequence type 156 (ST156) and carried the following antibiotic resistance genes: *tetM* (resistance to tetracycline) and *mefA* and *msrD* (resistance to macrolides). Genetic alterations in penicillin-binding protein (PBP) genes *pbp1a*, *pbp2a*, *pbp2x*, and *pbp2b* were consistent with those previously reported to be associated with nonsusceptibility to penicillin and matched the alterations in the multidrug-resistant clone Spain23F-ST81 (ATCC 700669, NC_011990) (13). Virulence genes, such as pneumolysin, autolysin, *pspA*, and *pspC*, and pilus gene *rrgC* were present in all three isolates. Although no variants associated with excessive virulence were identified, we cannot rule out strain-specific variation in virulence factor expression. Based on single nucleotide polymorphism (SNP) analysis, all three strains were highly similar and had only 1 SNP difference between the isolate from patient A and those from patients B and C. Comparison to 19 other UK serotype 9V ST156 pneumococcal isolates showed that the 3 outbreak strains clustered together (Fig. 2) and were, on average, 703 SNPs different from the other ST156 strains within the tree and 8,620 SNPs different from the reference strain S. pneumoniae R6, supporting the hypothesis that the 3 cases arose from a common source. Interestingly, four contemporaneous isolates that clustered with the outbreak strains (differed from outbreak isolates by 50 to 355 SNPs) (Fig. 2) had the same antibiotic resistance elements (*tetM*, *msrD*, and *mefA*), although they were from disparate regions and years (Table S1). Notably, the outbreak strains described here are from the same clonal lineage as the globally disseminated Pneumococcal Molecular Epidemiology Network (PMEN) clone Spain9V-3 (http://web1.sph.emory.edu/PMEN/index.html) (13, 14) but with a markedly different antimicrobial susceptibility pattern despite being of the same lineage. Strains from this lineage have previously been associated with an outbreak in a respiratory ward involving 6 patients over 10 days (15). The factors that facilitate pneumococcal transmission in the nosocomial setting are still unclear; however, in the outbreak reported herein, all patients had respiratory illnesses related to COPD or asthma at the time of exposure (Table 1) and would have been receiving treatment with nebulizers. It seems likely that respiratory droplet transmission may have allowed transmission to occur.

Serotype 9V is included in current pneumococcal vaccines, PCV13 and PPV23, and a single dose of PPV23 is recommended for adults over 65 year old (16), though uptake in the UK is only 69.8% (14). Patients in this outbreak were previously vaccinated (Table 1), but all received their vaccination more than 5 years prior to the outbreak.

The cases described were identified only because the isolates shared a similar pattern of antimicrobial resistance and because the presentations coincided. This outbreak, supported by WGS, highlights the potential brevity of the incubation period for invasive S. pneumoniae in susceptible hosts and emphasizes the possibility that nosocomial transmission events may be missed, particularly where affected patients have been readmitted to hospital with infections that are otherwise considered to be sporadic and community acquired.

## MATERIALS AND METHODS

### Bacterial isolates.

S. pneumoniae isolates from the cluster (*n* = 3) were tested for antimicrobial susceptibility using the Etest method (penicillin, tetracycline, erythromycin, ampicillin, cefotaxime, clindamycin, gentamicin, rifampin, teicoplanin, vancomycin, and moxifloxacin). Strains were referred to the Pneumococcal Reference Laboratory at Public Health England (PHE), Colindale, London, for serotyping by slide agglutination with standard antiserum (Statens Serum Institut, Copenhagen, Denmark) (17).

### DNA sequencing and genomic analysis.

Whole-genome sequencing (WGS) of the three cluster isolates was undertaken using Illumina HiSeq 2500 (Illumina, USA), and raw reads (reads submitted under PHE Pathogens BioProject PRJEB14267 at the European Nucleotide Archive; see Table S1 in the supplemental material) were assembled using SPAdes (18). MOST (19) was used to call multilocus sequence type (MLST) and SRST2 (20) was used for antibiotic resistance genes. Single nucleotide polymorphisms (SNPs) were called using BWA (21) and GATK (22) with acapsular S. pneumoniae R6 (NC_003098) used as a reference. Contextual serotype 9V ST156 isolates (Table S1) from the PHE archive were used for phylogenetic analysis.

## Supplementary Material

Supplemental material
