# Supplementary material for: Nosocomial Outbreak of Drug-Resistant Streptococcus pneumoniae Serotype 9V in an Adult Respiratory Medicine Ward
Source: J Clin Microbiol. 2017 Feb 22;55(3):776–82. doi: 10.1128/JCM.02405-16 (PMC5328445; doi:10.1128/JCM.02405-16)
Supplement: Supplemental material [file supp_55_3_776__index.html]

Nosocomial Outbreak of Drug-Resistant Streptococcus pneumoniae Serotype 9V in an Adult Respiratory Medicine Ward — Supplemental material 

# Nosocomial Outbreak of Drug-Resistant Streptococcus pneumoniae Serotype 9V in an Adult Respiratory Medicine Ward

## Supplemental material

- Supplemental file 1 -

  Table S1 (European Nucleotide Archive submission details for 9V ST156 clinical isolates used in SNP analysis)

  PDF, 143K
