# Supplementary material for: Nosocomial Outbreak of Drug-Resistant Streptococcus pneumoniae Serotype 9V in an Adult Respiratory Medicine Ward
Source: J Clin Microbiol. 2017 Feb 22;55(3):776–82. doi: 10.1128/JCM.02405-16 (PMC5328445; doi:10.1128/JCM.02405-16)
Supplement: Supplemental material [file JCM.02405-16_zjm999095371s1.pdf]

Supplementary Table 1. European Nucleotide Archive (ENA) submission details for 9V ST156 clinical isolates used in SNP analysis.

Sample ID	Accession No	Sero type	ST	Year of isolation	Age group	Source of SP isolation	Geo. location	Resistanc e genes	Antibiotic testing MIC (mg/L)									
									PEN	ERY	AMP	CTX	CLI	GEN	RIF	TEI	VAN	MFX
PHESPD0098	ERS1193396	9V	156	2003	83	blood	Devon	none	2 (I)	<=0.25 (S)	4 (R)	1 (I)	<=0.25 (S)	64 (R)	0.06 (S)	<=0.5 (S)	<=0.5 (S)	0.25 (S)
PHESPD0110	ERS1193408	9V	156	2003	58	pleural fluid	Wiltshire	none	1 (I)	<=0.25 (S)	2 (I)	0.5 (S)	<=0.25 (S)	32 (R)	0.06 (S)	<=0.5 (S)	<=0.5 (S)	0.25 (S)
PHESPD0141	ERS1193439	9V	156	2004	60	sputum	Cork, Ireland	none	R	S	ND	ND	ND	ND	S	ND	ND	ND
PHESPD0151	ERS1193449	9V	156	2005	< 1	ear	Hertford-shire	none	1 (I)	<=0.25 (S)	2 (I)	0.5 (S)	<=0.25 (S)	32 (R)	0.06 (S)	<=0.5 (S)	<=0.5 (S)	0.25 (S)
PHESPD0170	ERS1193468	9V	156	2006	51	sputum	Surrey	*tetM, ermB*	1 (I)	>16 (R)	2 (I)	0.5 (S)	>8.0 (R)	32 (R)	<=0.03 (S)	<=0.5 (S)	<=0.5 (S)	8 (R)
PHESPD0241	ERS1193539	9V	156	2009	7	csf	Lancashire	*tetM, msrD, mefA*	2 (I)	16 (R)	4 (R)	1 (I)	<=0.25 (S)	8 (R)	<=0.03 (S)	<=0.5 (S)	<=0.5 (S)	1 (R)
PHESPD0348	ERS1193646	9V	156	2012	33	other	London	*tetM, ermB*	1 (I)	>16 (R)	2 (I)	1 (I)	0.5 (S)	32 (R)	0.06 (S)	<=0.5 (S)	<=0.5 (S)	0.25 (S)
PHESPD0359	ERS1193657	9V	156	2012	70	blood	West Midlands	*tetM, msrD, mefA*	1 (I)	>16 (R)	4 (R)	1 (I)	<=0.25 (S)	32 (R)	0.06 (S)	<=0.5 (S)	0.5 (S)	0.5 (S)
PHESPD0370	ERS1193668	9V	156	2012	86	blood	West Midlands	none	2 (I)	<=0.25 (S)	4 (R)	1 (I)	<=0.25 (S)	32 (R)	0.06 (S)	<=0.5 (S)	<=0.5 (S)	2 (R)
PHESPD0372	ERS1193670	9V	156	2013	2	ear	London	*msrD, mefA*	2 (I)	16 (R)	4 (R)	1 (I)	<=0.25 (S)	64 (R)	0.06 (S)	<=0.5 (S)	<=0.5 (S)	1 (R)
PHESPD0391	ERS1193689	9V	156	2013	96	blood	Gloucesters hire	none	2 (I)	<=0.25 (S)	4 (R)	1 (I)	<=0.25 (S)	32 (R)	0.06 (S)	<=0.5 (S)	<=0.5 (S)	4 (R)
PHESPD0408	ERS1193706	9V	156	2013	91	blood	Lancashire	none	2 (I)	<=0.25 (S)	4 (R)	1 (I)	<=0.25 (S)	32 (R)	0.06 (S)	<=0.5 (S)	<=0.5 (S)	4 (R)

PHESPD0414	ERS1193712	9V	156	2013	73	blood	East Yorkshire	none	1 (I)	<=0.25 (S)	2 (I)	0.5 (S)	<=0.25 (S)	32 (R)	<=0.03 (S)	<=0.5 (S)	<=0.5 (S)	1 (R)
PHESPV0067	ERS1194236	9V	156	2009	3	blood	West Yorkshire	none	1 (I)	<=0.25 (S)	2 (I)	0.5 (S)	<=0.25 (S)	32 (R)	0.06 (S)	<=0.5 (S)	<=0.5 (S)	1 (R)
PHESPV0335	ERS1194503	9V	156	2013	54	blood	London	*tetM, ermB*	1 (I)	>16 (R)	4 (R)	0.5 (S)	>8.0 (R)	32 (R)	<=0.03 (S)	<=0.5 (S)	<=0.5 (S)	0.5 (S)
PHESPV0648	ERS1194816	9V	156	2014	72	blood	Lincoln-shire	*tetM, ermB*	2 (I)	>16 (R)	4 (R)	1 (I)	>8.0 (R)	64 (R)	<=0.03 (S)	<=0.5 (S)	<=0.5 (S)	0.5 (S)
PHESPV0661	ERS1194829	9V	156	2014	72	sputum	Cumbria	*tetM, msrD, mefA*	2 (I)	>16 (R)	4 (R)	1 (I)	<=0.25 (S)	32 (R)	0.06 (S)	<=0.5 (S)	<=0.5 (S)	8 (R)
PHESPV1188	ERS1195356	9V	156	2015	76	blood	London	*tetM, msrD, mefA*	2 (I)	>16 (R)	4 (R)	1 (I)	<=0.25 (S)	32 (R)	0.06 (S)	<=0.5 (S)	<=0.5 (S)	1 (R)
PHESPV1245	ERS1195413	9V	156	2015	76	sputum	London	*tetM, ermB*	2 (I)	>16 (R)	4 (R)	1 (I)	>8.0 (R)	32 (R)	0.06 (S)	<=0.5 (S)	<=0.5 (S)	>8.0 (R)
PHESPV1509	ERS1195673	9V	156	2015	60	blood	London	*tetM, msrD, mefA*	1 (I)	>16 (R)	2 (I)	0.25 (S)	<=0.25 (S)	32 (R)	0.06 (S)	<=0.5 (S)	<=0.5 (S)	1 (R)
PHESPV1510	ERS1195674	9V	156	2015	76	blood	London	*tetM, msrD, mefA*	1 (I)	>16 (R)	4 (R)	1 (I)	<=0.25 (S)	32 (R)	<=0.03 (S)	<=0.5 (S)	<=0.5 (S)	0.5 (S)
PHESPV1524	ERS1195688	9V	156	2015	73	sputum	London	*tetM, msrD, mefA*	2 (I)	>16 (R)	4 (R)	0.5 (S)	<=0.25 (S)	32 (R)	<=0.03 (S)	<=0.5 (S)	<=0.5 (S)	0.5 (S)

SP – *S. pneumoniae*, Geo. – geographical; S- susceptible; I – intermediate; R – resistant; ND – not done; PEN – penicillin; TET - tetracycline, ERY – erythromycin, AMP – ampicillin, CTX – cefotaximine, CLI – clindamycin, GEN – gentamicin, RIF – rifampicin, TEI – teicoplanin, VAN – vancomycin, MFX – moxifloxacin. S – susceptible, I – intermediate, R – resistant. EUCAST MIC breakpoints were used to interpret MIC results.
